# The Role of Next-Generation Sequencing in the Management of Asymptomatic, Young Slovenian Athletes for Distinction Between Athlete’s Heart and Cardiomyopathy

**DOI:** 10.3390/biomedicines14071505

**Published:** 2026-07-02

**Authors:** Špela Stangler Herodež, Eva Čokolič, Nik Slavic, Tomaž Podlesnikar, Matjaž Vogrin, Nadja Kokalj Vokač, Danijela Krgović

**Affiliations:** 1 Clinical Institute of Genetic Diagnostics, University Medical Centre Maribor, 2000 Maribor, Slovenia; 2 Department of Molecular Biology, Faculty of Medicine, University of Maribor, 2000 Maribor, Slovenia; 3 Department of Cardiac Surgery, University Medical Centre Maribor, 2000 Maribor, Slovenia; 4 Department of Orthopaedics, University Medical Centre Maribor, 2000 Maribor, Slovenia; 5 Institute of Sports Medicine, Faculty of Medicine, University of Maribor, 2000 Maribor, Slovenia

**Keywords:** athlete’s heart, cardiomyopathy, sudden cardiac death, sport, next-generation sequencing

## Abstract

**Background**: Intensive, prolonged endurance and strength training in apparently healthy young athletes causes many physiologic and structural adjustments in the heart, known as athlete’s heart. The correct distinction between physiological adaptations and pathological changes is crucial for the athlete, therefore screening programs are recommended by medical and sports associations. Nevertheless, a small but not negligible proportion of young competitive athletes die by sudden death. In 25% the cause of sudden cardiac death is genetic. Determining the genetic component is of key importance for distinction between athlete’s heart and cardiomyopathy. **Methods**: A gene panel next-generation sequencing (NGS) was performed in 41 apparently healthy young athletes without previously known heart disease. **Results**: Across 174 genes with known associations to different cardiac conditions, we identified nine variants: two pathogenic (P) variants and seven variants of unknown significance (VUSs). For all of them a more detailed follow-up with a cardiologist was advised. **Conclusions**: The results in our study suggest that targeted sequencing of genes associated with cardiovascular disease is the key that enables correct differentiation between athlete’s heart and cardiomyopathy, leading to fast and accurate clinical intervention where necessary. In this way, initial pathological changes are not confused with physiological ones, which could be fatal for the athlete if they continue with competitive sport.

## 1. Introduction

Intensive and long-term physical training induces a spectrum of structural, functional, and electrical cardiac adaptations commonly referred to as athlete’s heart. These changes include left ventricular (LV) wall thickening, increased chamber dimensions, and electrocardiographic alterations, which may substantially overlap with the phenotypic expression of inherited cardiomyopathies, particularly in young individuals with high training loads [1]. This overlap creates a diagnostic challenge, particularly in asymptomatic athletes, in whom physiological changes and early-stage cardiomyopathy may be clinically indistinguishable using standard diagnostic tools.

Cardiomyopathies are usually genetically determined. They are primarily inherited in an autosomal dominant pattern and involve mutations in several different genes that disrupt critical structures in heart muscle cells, primarily the sarcomeres (contractile units), cytoskeleton (cellular scaffolding), or desmosomes (intercellular junctions), ultimately impairing the heart’s ability to pump blood [2]. The genetic background is deeply tied to specific disease types. Originally, only the following cardiomyopathies were considered:

- Hypertrophic Cardiomyopathy (HCM): primarily caused by mutations in sarcomeric genes (most notably *MYH7* and *MYBPC3*), which make the heart muscle abnormally thick and prone to high contractility [3].
- Dilated Cardiomyopathy (DCM): frequently caused by mutations in cytoskeletal and nuclear membrane genes, with truncating variants in the *TTN* and *LMNA* genes being the most common, which lead to enlarged, weakened ventricles [2].
- Arrhythmogenic Cardiomyopathy (ACM): typically driven by mutations in desmosomal genes (such as *PKP2*), which cause heart muscle to be replaced by fat and scar tissue, leading to severe arrhythmias [2].

Dysfunction can also be electrical, so disorders affected by electrical dysfunction without a structural substrate can also be considered cardiomyopathies (e.g., channelopathies and ryanodine receptors). Because clinical severity and age of onset vary even among family members with the exact same mutation, environmental factors and additional genetic modifiers play a significant role in how the disease physically manifests [3].

Genomic analyses have significantly advanced the understanding of inherited cardiovascular diseases and their clinical management [4]. According to the 2023 European Society of Cardiology (ESC) guidelines, genetic testing and counseling are recommended in patients with cardiomyopathy when results are expected to provide clinically actionable information, particularly when pathogenic or likely pathogenic variants are identified [5]. For an increasing number of inherited cardiac conditions, a genetic diagnosis provides prognostic information and may directly influence therapeutic strategies, family screening, and long-term surveillance [6,7]. However, a substantial gap remains between the growing complexity of next-generation sequencing (NGS) data and its optimal integration into everyday clinical decision-making [4,6].

Sudden cardiac death (SCD) in young athletes, although relatively rare, represents a catastrophic clinical event and may be the first manifestation of an underlying inherited cardiac disorder [8]. Epidemiological studies have demonstrated that cardiomyopathies and inherited arrhythmia syndromes are among the most common causes of SCD in the population of young athletes, where they often occur in individuals without prior symptoms or known structural heart disease [9,10]. The *DES* gene, which encodes desmin, a muscle-specific protein, is for example known as a gene whose mutations cause SCD. Mutations (like p.A120D) disrupt desmin filament assembly, causing protein aggregation and preventing localization at the cardiac intercalated disk. This structural failure disrupts electrical and mechanical stability, triggering severe arrhythmias and SCD [11]. SCD-causing genes generally fall into two categories: structural cardiomyopathies (e.g., *MYH7*, *MYBPC3*, *TTN*, *LMNA*, *DSP*, *PKP2*, *JUP*) and primary electrical disorders (channelopathies) (e.g., *SCN5A*, *KCNQ1*, *KCNH2*, *RYR2*) [12].

These findings highlight the limitations of symptom-based assessment and conventional preparticipation screening strategies.

Clinical phenotyping remains the cornerstone of cardiovascular evaluation in athletes with suspected heart disease. In accordance with the current guidelines, standard diagnostic approaches include resting and ambulatory electrocardiography, echocardiography, exercise testing, and cardiac magnetic resonance imaging [5]. Nevertheless, several inherited cardiomyopathies may present with subtle, borderline, or even normal findings in early disease stages, particularly in physically active individuals [6,7]. Furthermore, exercise itself may modulate disease expression and arrhythmic risk, especially in Arrhythmogenic Cardiomyopathies, complicating phenotypic interpretation [13,14]. As a result, a subgroup of athletes remain in a “diagnostic gray zone”, where neither physiological adaptation nor pathological remodeling can be confidently confirmed or excluded.

NGS-based genetic testing has emerged as a valuable adjunct in the evaluation of inherited cardiovascular diseases. Targeted gene panels and broader sequencing approaches enable the identification of disease-associated variants implicated in cardiomyopathies and channelopathies [4,6]. The clinical validity of gene–disease associations has been systematically assessed through evidence-based frameworks, such as those developed by the Clinical Genome Resource (ClinGen), providing important guidance for variant interpretation [7]. Nevertheless, the identification of a genetic variant alone is insufficient; accurate classification and careful integration with clinical phenotype are essential to avoid misdiagnosis and inappropriate management [15].

Importantly, malignant ventricular arrhythmias may represent an early or primary manifestation of hereditary cardiomyopathies that lead to the development of overt structural abnormalities [6,8]. Arrhythmogenic genotypes have been identified in familial Dilated Cardiomyopathy and other inherited conditions, underscoring the role of genetic predisposition in arrhythmic risk stratification [8]. Such genotype–phenotype relationships are particularly relevant in young and asymptomatic individuals, including athletes, in whom traditional risk markers may be absent.

In the context of competitive sports, the integration of genetic data with comprehensive clinical phenotyping may provide incremental diagnostic and prognostic value. Identification of a pathogenic variant may allow differentiation between athlete’s heart and inherited cardiomyopathy, influence decisions regarding sports participation, determine individualized follow-up strategies, and enable cascade genetic testing of family members [5,7]. Conversely, the absence of disease-causing variants, may increase confidence in a diagnosis of physiological cardiac remodeling in terms of athlete’s heart.

In this article, we examine the role of NGS in the management of asymptomatic young athletes from the north-eastern part of Slovenia for distinction between athlete’s heart and cardiomyopathy. We also discuss the clinical implications of incorporating genotypic information into contemporary diagnostic and risk stratification pathways.

## 2. Materials and Methods

### 2.1. Athletes

Included were 41 apparently healthy young athletes (30 males, 11 females) without previously known heart disease who came for a routine check-up in the frame of a screening program performed every second year. The screening program consisted of the athlete’s personal and family health history, physical examination, spirometry, a 12-lead resting electrocardiogram (ECG) and blood sampling for a blood count. The athletes were between 15 and 35 years old and trained in football, triathlon and swimming.

Athletes signed written informed consent for the use of their personal and family medical history, screening program results, and genetic testing reports.

### 2.2. Next-Generation Sequencing (NGS)

NGS was performed using the genomic DNA isolated from the peripheral blood with the QIAamp DNA Blood Mini Kit (QIAGEN, Hilden, Germany). DNA library construction for genetic profiling of 174 genes with known associations to 17 different inherited cardiac conditions (ICCs) and predefined metabolic cardiovascular disorders associated with premature coronary artery disease, such as familial hypercholesterolemia, was performed with the Illumina TruSight Cardio Sequencing Kit (Illumina, San Diego, CA, USA) according to the manufacturer’s instructions [16]. The prepared DNA libraries were then sequenced on the Illumina MiSeq platform (151 bp paired-end reads) (Illumina, San Diego, CA, USA) with an average depth coverage of 300× and an average output of 15 Gb per sample.

Analysis of the sequencing data was carried out using the MiSeq Reporter software version 2.5.42.5 [16], according to the BWA Enrichment workflow. Analysis and interpretation of variants obtained in the VCF file were carried out the commercially available bioinformatics tool Franklin (QIAGEN, Hilden, Germany), which was also used for interpretation of genetic variants and partial literature search.

Classification of genetic variants was performed considering the following criteria: classification as pathogenic (P), likely pathogenic (LP) or variant of unknown significance (VUS) according to ACMG/AMP guidelines [17,18]; allele frequency <10^−5^ in the genome aggregation database (gnomAD v4.1); computational evidence supporting a deleterious effect based on in silico prediction tools; review of the relevant published literature. The ClinVar database was last accessed on 15 March 2025. Where applicable, ClinGen gene–disease validity frameworks and gene-specific expert panel curation guidelines were utilized to support the classification. To ensure interpretation reliability, all variants were independently reviewed by at least two expert geneticists, and any discrepancies were resolved by consensus.

## 3. Results

**Figure 1:**
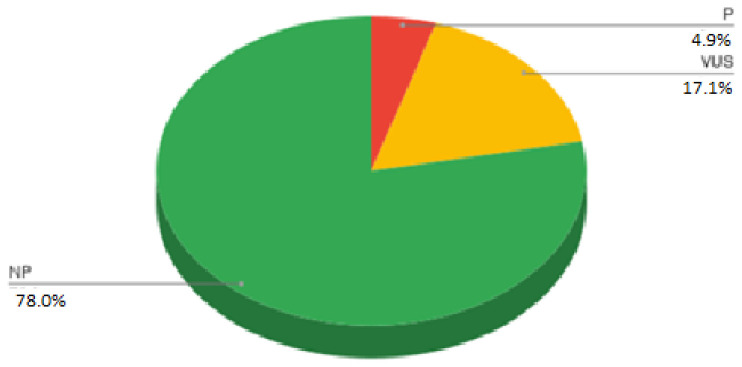
Results of 41 (100%) tested athletes: 2 (4.9%) pathogenic cases (Ps), 7 (17.1%) VUSs and 32 (78%) non-pathogenic cases (NPs).

The results of 41 NGS cases followed by targeted bioinformatics revealed two pathogenic (P) (Table 1) and seven VUS (Table 2) variants. The remaining 32 cases had non-pathogenic variants (NPs) in any of the 174 genes tested (Figure 1). Closer follow-up with a cardiologist was recommended for all P and VUS cases.

### 3.1. Pathogenic Variants (Athletes 1–2)

#### 3.1.1. Athlete 1

A 15-year-old female triathlete carried a frameshift insertion c.80478dupA, which creates a premature translational stop signal (p.(Trp26827MetfsTer6)) in the A band of the *TTN* gene; truncation variants in this region are significantly associated with an increased risk of Dilated Cardiomyopathy [19]. The girl was clinically asymptomatic, had no laboratory abnormalities, and had a normal electrocardiogram (ECG). Her family history of cardiovascular disease was negative. No structural cardiac abnormalities were detected during follow-up.

#### 3.1.2. Athlete 2

A 30-year-old male, football player, with abnormal laboratory findings (elevated LDL, low-density lipoprotein cholesterol), normal 12-lead ECG and positive family history for hypercholesterolemia had a missense variant c.81C>G in the *LDLR* gene. This variant is present in the ClinVar database (Variation ID 226304) and is predicted to be deleterious to protein function as it breaks down one of the disulfide bonds [20,21,22]. In the literature, this variant is widely reported as a pathogenic variant found mainly in familial hypercholesterolemia (FHCL) patients of European origin [23,24,25,26,27].

### 3.2. Variants of Uncertain Significance (Athletes 3–9)

#### 3.2.1. Athlete 3

In a 27-year-old male football player with no laboratory or ECG deviations and a negative family history (−FH), NGS genetic screening revealed the presence of the c.1000G>A VUS variant in the *JPH2* gene. The glycine at codon 334 is replaced by amino acid serine with similar properties. This amino acid position is conserved. In silico analysis predicts that this alteration is deleterious. This variant is present in the ClinVar database (Variation ID 4614297) as a VUS and has not been reported in the literature. However, missense variants in *JPH2* are reported to be associated with a risk for Hypertrophic Cardiomyopathy 17 (CMH17; OMIM#613873).

#### 3.2.2. Athlete 4

A 19-year-old male football player with no significant clinical laboratory or ECG findings and a negative FH (−FH) was found to carry a VUS variant c.5294G>A in the *MYH11* gene. The amino acid arginine at codon 1758 is replaced by glutamine with highly similar properties. This variant is present in the ClinVar database (Variation ID 161316). In the literature this missense change has been reported in multiple individuals with thoracic aortic aneurysm and dissection (TAAD) [28,29].

#### 3.2.3. Athlete 5

A 28-year-old male football player with a positive family history for hypertriglyceridemia (+FH), without laboratory deviations and normal ECG findings had a frameshift insertion c.732dup, which creates a premature translational stop signal in the *CREB3L3* gene. This variant is present in the ClinVar database (Variation ID 4614297) as a VUS. In the literature it has been reported in individuals affected with hypertriglyceridemia 2 as a heterozygous genotype in familial pedigrees and in unaffected individuals [30,31,32]. The literature also states that individuals with severe hypertriglyceridemia were found to be 20× more likely to carry a heterozygous loss-of-function (LOF) variant in *CREB3L3* [33]. According to current classification criteria, the variant was categorized as a VUS, with a possibility of pathogenicity.

#### 3.2.4. Athlete 6

In a 23-year-old female swimmer with a significant ECG deviation showing atrial fibrillation (AF), normal laboratory results and unremarkable family history, genetic testing identified missense variant c.1331A>T in the *HCN4* gene, where valine replaces aspartic acid at codon 444 of the HCN4 protein (p.(Asp444val)). Missense variants in *HCN4* are reported to be associated with a risk for sick sinus syndrome 2 (SSS2; OMIM#163800). The identified variant is present in the ClinVar database (Variation ID 1349052) as a VUS and has not been reported in the literature.

#### 3.2.5. Athlete 7

A 25-year-old male football player with no laboratory or ECG abnormalities and a negative FH (−FH) was identified as a carrier of a VUS in the *KCNQ1* gene. c.328G>A is a missense variant predicted to cause replacement of valine by isoleucine at amino acid 110 (p.(Val110Ile)) and has been observed in individuals with Long QT syndrome [34,35,36]. It is also present in the ClinVar database (Variation ID: 53034). While experimental studies have shown that this missense change affects function of the KCNQ1 protein [36,37], per current criteria, the variant was classified as VUS.

#### 3.2.6. Athlete 8

A 32-year-old male football player with no laboratory deviations, normal ECG, and a negative FH (−FH) carried a missense variant in the *MYH6* gene. The c.2161C>T variant replaces basic and polar arginine with neutral and slightly polar tryptophan at codon 721 (p.(Arg721Trp)). This missense change is present in population databases (gnomAD v4.1.0: 0.03%) and has been observed in individuals with atrial fibrillation, coarctation of the aorta and/or sick sinus syndrome [38,39,40]. The identified variant is present in the ClinVar database (Variation ID 25717017) as a VUS. Experimental studies have also shown that c.2161C>T missense change affects functioning of the MYH6 protein [41].

#### 3.2.7. Athlete 9

Missense variant in *DTNA* was identified in a 22-year-old male triathlete who presented with a negative FH (−FH), normal laboratory values and an ECG deviation that revealed atrial fibrillation (AF). The c.177A>G alteration replaces an amino acid at position 59, where isoleucine is replaced with methionine (p.(Ile59Met)). This missense change is rare (gnomAD v4.1.0: 0.004%) and was classified as a VUS.

## 4. Discussion

Inherited cardiomyopathies and channelopathies display substantial phenotypic heterogeneity and can significantly overlap with physiological structural and functional adaptations, commonly referred to as athlete’s heart. This overlap creates a “diagnostic gray zone”, making clinical evaluation and variant interpretation a challenge, particularly in asymptomatic athletes. NGS has emerged as a valuable tool for identifying disease-associated variants and distinguishing true pathological changes from benign, training-induced cardiac remodeling.

In current screening programs for athletes, genetic testing as a screening strategy remains relatively limited in availability and accessibility compared with conventional diagnostic modalities and is generally restricted to specific situations in which a genetic disease is highly suspected. Although NGS is a diagnostic tool with considerable potential, its routine implementation requires a structured clinical approach that includes pre- and post-test genetic counseling. This is especially important in cases in which deviations are found that may lead to potential psychological consequences, especially in asymptomatic athletes, since it is known that pathogenic variants do not always lead to phenotypic changes due to, e.g., incomplete penetrance—a challenge even more pronounced in the case of VUSs [5]. Pre-test genetic counseling is paramount and must explicitly prepare the asymptomatic athlete for the possibility of uninformative or ambiguous results, such as VUSs, as well as secondary incidental findings. It must address the potential psychological burden, impact on family members, and implications for athletic eligibility. Post-test counseling focuses on translating complex genetic data into clear, actionable clinical context. This is critical because pathogenic (P) variants do not always manifest phenotypically due to incomplete penetrance, which can provoke substantial anxiety in clinically healthy athletes [5]. Only such a strict approach reduces unnecessary psychological distress and prevents unjustified exclusion from competitive sports.

Comprehensive diagnostic approaches—merging genomic data with continuous cardiovascular evaluation, athlete demographics and sporting discipline—are increasingly essential for refining risk stratification. The present cohort illustrates these complexities across three major outcome groups: pathogenic variants (Table 1), VUSs (Table 2) and the majority of athletes with no disease-associated variants. These findings provide critical context for how targeted sequencing can facilitate fast and accurate clinical intervention, ensuring that initial pathological changes are not fatally confused with physiological adaptations of the heart.

### 4.1. Athletes with P Variants

In our cohort of 41 asymptomatic young athletes, NGS identified two variants classified as pathogenic according to the current ACMG/AMP guidelines [17,18]. In accordance with international consensus, it is important to point out that it is not enough to just look at the gene, but we must also relate the underlying genetic variant to the athlete’s demographics, clinical presentation and the specific physical demands of their discipline. In accordance to the ESC guidelines evidence-based longitudinal follow-up is essential for identified pathogenic cases [5].

#### 4.1.1. Athlete 1—*TTN*

The identification of a pathogenic frameshift variant in the *TTN* gene (c.80478dupA) in a 15-year-old triathlete presents a significant clinical challenge. Titin-truncating variants (TTNtvs) represent the most common genetic cause of Dilated Cardiomyopathy (DCM), though their clinical penetrance is frequently incomplete and highly influenced by environmental “second hits,” such as intensive endurance exercise [42]. While this patient currently presents as asymptomatic with a normal baseline screening, the high-intensity nature of triathlon training, characterized by sustained high cardiac output, represents a relevant physiological factor that could influence phenotypic expression over time.

Furthermore, the segregation of the variant from the asymptomatic mother confirms an autosomal dominant inheritance pattern with variable expressivity (Figure S1). As noted in the current literature, the absence of symptoms in a parent does not rule out future development in the offspring, especially when the physiological cardiac load differs significantly [43]. Consequently, this finding highlights the value of NGS in identifying at-risk individuals before the onset of overt cardiac remodeling, justifying long-term, non-invasive sports-cardiology monitoring based on the genetic susceptibility [43].

#### 4.1.2. Athlete 2—*LDLR*

While the primary focus of this study was the differentiation of athlete’s heart and structural cardiomyopathy, the inclusion of metabolic targets in our NGS panel proved important for identifying non-structural cardiovascular risks. The case of a 30-year-old football player with a pathogenic *LDLR* variant (c.81C>G) underscores the importance of screening for familial hypercholesterolemia (FHCL) within athletic populations. Athletes are often perceived as the “gold standard” of health, yet they remain susceptible to underlying genetic metabolic disorders that can elevate the risk of premature coronary artery disease (CAD).

Our findings demonstrate that relying solely on physical fitness and standard fitness metrics can mask significant genetic cardiovascular risks. In the context of an asymptomatic athlete, the identification of an *LDLR* pathogenic variant provides a crucial opportunity for early primary prevention and lipid-lowering strategies before advanced atherosclerotic changes occur. This case illustrates that low-tier genetic screening can successfully unmask silent metabolic liabilities in highly fit individuals who would otherwise be classified as low risk by traditional clinical scoring tools [44].

### 4.2. Athletes with VUSs

In our cohort of 41 asymptomatic young athletes, NGS identified seven variants classified as VUSs, according to the current ACMG/AMP guidelines [17], in the *JPH2*, *MYH11*, *CREB3L3*, *HCN4*, *KCNQ1*, *MYH6* and *DTNA* genes. In strict accordance with international consensus, we emphasize that these VUS findings must not be utilized for definitive clinical decision-making, predictive genetic testing of asymptomatic relatives, or to justify sports disqualification. Premature clinical action or career restriction based solely on a VUS can lead to unwarranted psychological distress and unnecessary medicalization in healthy young athletes.

Nevertheless, within the framework of sports cardiology, reporting these seven VUSs holds substantial exploratory and long-term diagnostic value, particularly when differentiating between physiological cardiac adaptation (“athlete’s heart”) and early, phenotype-negative cardiomyopathy. In cases in which an athlete presents with a borderline or “diagnostic gray zone” clinical phenotype—such as mild concentric left ventricular hypertrophy or ambiguous electrocardiographic abnormalities—the presence of a VUS in a highly relevant cardiac gene (e.g., *KCNQ1* or *HCN4*) serves as a phenotypic “red flag”. Rather than altering clinical management based on the genetic variant itself, these findings justify standard, non-invasive longitudinal sports-cardiology surveillance (e.g., routine echocardiography or exercise ECG) driven primarily by the borderline clinical presentation.

Furthermore, documenting these variants is crucial for the ongoing evolution of genomic medicine. Many variants in cardiomyopathy and channelopathy genes are unique to specific families (private mutations), initially lacking the statistical power in global databases required for a pathogenic classification. To resolve the uncertainty of these seven VUSs, future steps should focus on diagnostic family co-segregation analyses—testing affected or older family members to track the variant with the disease phenotype—rather than clinical cascade screening of healthy individuals. Over time, as more segregation and functional data accumulate, these VUSs may be systematically upgraded or downgraded, ultimately refining risk stratification for future generations of athletes.

#### 4.2.1. Athlete 3—*JPH2*

The identification of a missense VUS in the *JPH2* gene (c.1000G>A) in a 27-year-old football player highlights the “diagnostic gray zone” inherent in sports cardiology. Junctophilin-2 is critical for the structural integrity of T-tubules and calcium handling [45]. Variants in this gene are associated with Hypertrophic Cardiomyopathy (HCM) [46]. Specifically, JPH2 stabilizes the junctional membrane complex (JMC), and its disruption can lead to perturbed Ca2+ dynamics—a hallmark of both HCM and heart failure [47,48].

In this athlete, the normal resting ECG suggests that the variant has not resulted in clinical disease expression. The primary challenge in such cases lies in the long-term differentiation between early-stage, phenotype-negative genetic cardiomyopathy and benign physiological left ventricular hypertrophy common in high-intensity field sports [49]. Rather than guiding immediate changes in athletic eligibility or intervention, this VUS serves as an exploratory biomarker. It underscores the necessity of standard periodic sports-cardiology re-evaluations and genomic re-classification as global database evidence evolves [50].

#### 4.2.2. Athlete 4—*MYH11*

The detection of a VUS in the *MYH11* gene (c.5294G>A) in a 19-year-old football player presents the clinical challenge of managing incidental genomic findings in asymptomatic athletes. *MYH11* encodes smooth muscle myosin heavy chain, and pathogenic variants are typically associated with Familial Thoracic Aortic Aneurysm and Dissection (FTAAD) [50]. While the athlete is currently asymptomatic, whether the hemodynamic stress of competitive football promotes vascular fatigue in this genetic context remains speculative.

Since this finding is a VUS, it cannot be used for definitive clinical decision-making or immediate disqualification [50]. However, given the theoretical risk, our dataset supports adopting a proactive, localized surveillance strategy. Based on the cardiovascular evaluation parameters available in our cohort, we recommend serial echocardiographic monitoring of the aortic root and ascending aorta to track any structural changes over time [51]. This approach emphasizes the value of integrating genomic data with ongoing clinical phenotyping to safeguard athlete health.

#### 4.2.3. Athlete 5—*CREB3L3*

The identification of a heterozygous variant in *CREB3L3* (c.732dup) in a 28-year-old football player with a positive family history (+FH) highlights the clinical relevance of exploring metabolic genetics alongside athletic cardiovascular risk. *CREB3L3* encodes a membrane-bound transcription factor essential for triglyceride metabolism, and dysfunctional variants are associated with hypertriglyceridemia, which can elevate the long-term risk for premature coronary artery disease (CAD) [52].

In this context, the diagnostic challenge extends beyond structural cardiac remodeling to potential metabolic factors influencing long-term vascular health. Although the athlete’s ECG and baseline clinical findings are currently normal, identifying this genetic variant underscores the utility of NGS in identifying subclinical metabolic predispositions [32]. This finding supports the integration of regular metabolic screening within the athlete’s routine clinical follow-ups and highlights the potential role of family co-segregation analysis in first-degree relatives to better characterize the variant’s pathogenicity.

#### 4.2.4. Athlete 6—*HCN4*

The identification of a heterozygous *HCN4* variant (c.1331A>T) in a 23-year-old female athlete with atrial fibrillation (AF) highlights a complex intersection between cardiac electrical remodeling and genetic predisposition. HCN4 is the primary molecular component of the “funny” current (I_f_) responsible for sinoatrial node pacemaker activity. Although pathogenic variants are classically associated with sick sinus syndrome 2 (SSS2), they are increasingly recognized for predisposing individuals to atrial arrhythmias [53]. In this case, the clinical evaluation involves differentiating physiological exercise-induced bradycardia from pathological sinus node dysfunction, as high-intensity training may interact with an underlying *HCN4* substrate [54]. This finding underscores the utility of NGS in identifying potential electrical predispositions in athletes presenting with arrhythmias. It supports the value of long-term electrocardiographic surveillance and clinical phenotyping to monitor for chronotropic competence, while also highlighting the role of family co-segregation analysis in first-degree relatives to better characterize the variant’s clinical significance.

#### 4.2.5. Athlete 7—*KCNQ1*

The detection of a heterozygous *KCNQ1* variant (c.328G.A) in an asymptomatic 25-year-old football player presents a diagnostic challenge, as the gene’s role in the slow delayed rectifier potassium (I_Ks_) current is critical for cardiac repolarization. Pathogenic variants in this gene are classically associated with Long QT Syndrome Type 1 (LQT1), in which cardiac events are frequently triggered by the sympathetic surge of physical exertion, though they can also predispose individuals to familial atrial fibrillation [55]. In a competitive athlete, this finding is particularly relevant because potential repolarization abnormalities might not be apparent on a resting ECG [56]. Identifying this variant underscores the value of incorporating NGS data alongside routine clinical phenotyping, such as exercise stress testing, to evaluate dynamic cardiac responses. This integrated approach aids in characterizing subclinical electrical phenotypes and supports the consideration of family co-segregation analysis in first-degree relatives to further clarify the variant’s clinical significance.

#### 4.2.6. Athlete 8—*MYH6*

The identification of a heterozygous *MYH6* variant (c.2161C>T) in an asymptomatic 32-year-old football player presents a diagnostic challenge, as this gene encodes the α-myosin heavy chain (α-MHC) essential for sarcomere contraction and atrial development. While currently phenotype-negative, pathogenic variants in *MYH6* are associated with Hypertrophic Cardiomyopathy 14 (CMH14) and Dilated Cardiomyopathy 1EE (CMD1EE), demonstrating significant phenotypic plasticity [57,58]. In an elite athlete, the chronic hemodynamic stress of football could potentially act as an environmental trigger for late-onset remodeling, which might initially be misidentified as physiological “athlete’s heart” [9]. Understanding the precise clinical trajectory of such asymptomatic variants in athletic populations requires further longitudinal clinical data. Continued observation and a better understanding of family co-segregation analysis in first-degree relatives in these specific genetic profiles remain important areas for future research to clarify variant pathogenicity [54].

#### 4.2.7. Athlete 9—*DTNA*

The identification of a heterozygous *DTNA* variant (c.177A>G) in a 22-year-old football player with atrial fibrillation (AF) highlights a critical diagnostic intersection. *DTNA* encodes dystrobrevin-alpha, a component of the dystrophin-associated protein complex essential for the structural integrity of the sarcolemma and signal transduction; pathogenic variants are primarily associated with Left Ventricular Noncompaction 1 (LVNC1) [59]. In an elite athlete, the diagnostic distinction between physiological and pathological features is particularly complex, as high-intensity training can induce prominent trabeculations that mimic the pathological noncompaction criteria seen in LVNC [60]. In this case, the presence of AF may represent an early electrical manifestation of an underlying substrate. Tracking the long-term clinical trajectory of such phenotype-negative individuals remains a subject for further longitudinal research. Furthermore, accumulating data through family cascade screening could help clarify the precise clinical significance and pathogenicity of this variant.

## 5. Conclusions

This study demonstrates the feasibility and potential clinical utility of the NGS genetic testing strategy within a cohort of 41 asymptomatic young athletes. NGS technology can serve as a valuable complementary bridge between molecular data and clinical practice. In the challenging diagnostic overlap between athlete’s heart and cardiomyopathy, targeted sequencing provides exploratory molecular insights that may assist clinicians in evaluating benign adaptations alongside early pathological markers. However, genetic data alone cannot definitively differentiate routine remodeling from cardiomyopathy and must always be integrated with comprehensive clinical phenotyping to prevent potential mismanagement.

It would be worthwhile to continue the study with a group of athletes who fall within the “diagnostic gray zone”, where the distinction between physiological and pathological cardiac remodeling is still uncertain, using standard assessment protocols. Focusing on this subgroup of athletes could provide additional insights into the diagnostic yield and clinical utility of genetic testing in daily practice decision-making. At the same time, the benefits of the method used could be better demonstrated with a larger study.

Risk stratification in competitive sports is a multi-dimensional process that requires the merging of several distinct factors. It is not enough to look at a gene; we must integrate the underlying genetic variant with the athlete’s demographics, clinical presentation, and the specific physical demands of their discipline. We conclude that, for the identified pathogenic cases, evidence-based longitudinal follow-up is essential, whereas for VUS cases, standard non-invasive sports-cardiology monitoring should be tailored primarily to the athlete’s clinical phenotype rather than to the genetic result alone. By adopting this multifaceted model and strictly adhering to ACMG guidelines regarding VUS uncertainty, we can better evaluate the cardiovascular safety of young athletes without imposing unnecessary or premature ends to their professional careers.

On the other hand a limitation of this study is also the lack of systematic, comprehensive cardiovascular phenotyping such as routine echocardiography, cardiac magnetic resonance imaging (CMR), exercise stress testing, or Holter monitoring for the entire cohort. Because our screening program relied on standard baseline metrics (ECG and clinical history), we cannot fully correlate the genetic findings (particularly the seven VUSs) with subtle structural or arrhythmic features of athlete’s heart versus early-stage cardiomyopathy. Future longitudinal studies incorporating advanced imaging alongside NGS are strictly necessary to evaluate the true clinical utility of genetic testing in the “diagnostic gray zone” of sports cardiology.

## Figures and Tables

**Table 1:** Athletes with pathogenic (P) variants.

Athlete	Gender	Age (Years)	Sport	Laboratory Deviation	ECG Deviation	FH	Gene	HGVS (cDNA/Protein)	Zygosity	Variant Type	ACMG/ACGS Criteria
1	F	15	triathlon	NO	NO	−	*TTN*	c.80478dupA p.(Trp26827MetfsTer6)	het	frameshift insertion	PVS1_vstr, PM2_sup, PS4_mod
2	M	30	football	YES (LDL)	NO	+	*LDLR*	c.81C>G p.(Cys27Trp)	het	missense	PM1-mod, PM2-sup, PM5_mod, PP3_sup, PP4_sup, PS3-mod, PS4_str

Abbreviations: F, female; M, male; LDL, low-density lipoprotein; ECG, electrocardiogram; FH, family history; HGVS, Human Genome Variation Society; het, heterozygous; P, pathogenic.

**Table 2:** Athletes with variants of uncertain significance (VUSs).

Athlete	Gender	Age (Years)	Sport	Laboratory Deviation	ECG Deviation	FH	Gene	HGVS (cDNA/Protein)	Zygosity	Variant Type	ACMG/ACGS Criteria
3	M	27	football	NO	NO	−	*JPH2*	c.1000G>A p.(Gly334Ser)	het	missense	PM2_sup, PP3_mod
4	M	19	football	NO	NO	−	*MYH11*	c.5294G>A p.(Arg1758Gln)	het	missense	PM2_sup, PP3_mod
5	M	28	football	NO	NO	+	*CREB3L3*	c.732dup p.(Lys245GlufsTer130)	het	frameshift insertion	PVS1_str, PM2_sup
6	F	23	swimming	NO	YES (AF)	−	*HCN4*	c.1331A>T p.(Asp444Val)	het	missense	PM2_sup, PP3_mod
7	M	25	football	NO	NO	−	*KCNQ1*	c.328G>A p.(Val110Ile)	het	missense	PM1_mod, PM2_sup
8	M	32	football	NO	NO	−	*MYH6*	c.2161C>T p.(Arg721Trp)	het	missense	PM2_sup, PM5_mod
9	M	22	triathlon	NO	YES (AF)	−	*DTNA*	c.177A>G p.(Ile59Met)	het	missense	PM2_sup, PP3_sup

Abbreviations: F, female; M, male; ECG, electrocardiogram; FH, family history; het, heterozygous; AF, atrial fibrillation; VUS, variant of uncertain significance.

## Data Availability

The original contributions presented in this study are included in the article/Supplementary Materials. Further inquiries can be directed to the corresponding author.

## Supplementary Materials

The following supporting information can be downloaded at https://www.mdpi.com/article/10.3390/biomedicines14071505/s1: Figure S1: Sanger sequencing.

## Abbreviations

The following abbreviations are used in this manuscript:

α-MHC	α-Myosin heavy chain
ACC	American College of Cardiology
AF	Atrial fibrillation
AHA	American Heart Association
ATFB3	Familial atrial fibrillation 3
BSA	Body surface area
CAC	Coronary artery calcium
CAD	Coronary artery disease
CCTA	Coronary CT angiography
ClinGen	Clinical Genome Resource
CMD1EE	Dilated cardiomyopathy 1EE
CMH14	Hypertrophic cardiomyopathy 14
CMR	Cardiovascular magnetic resonance
DCM	Dilated cardiomyopathy
ECG	Electrocardiogram
ESC	European Society of Cardiology
FH	Family history
+FH	Positive family history
−FH	Negative family history
FHCL	Familial hypercholesterolemia
FTAAD	Familial thoracic aortic aneurysm and dissection
HCM	Hypertrophic cardiomyopathy
HYTG2	Hypertriglyceridemia 2
ICCs	Inherited cardiac conditions
If	“Funny” current
IKs	Slow delayed rectifier potassium current
JMC	Junctional membrane complex
LGE	Late gadolinium enhancement
LQT1	Long QT syndrome type 1
LV	Left ventricular
LVH	Left ventricular hypertrophy
LVNC1	Left ventricular noncompaction 1
NGS	Next-generation sequencing
NP	Non-pathogenic
P	Pathogenic
SCD	Sudden cardiac death
SSS2	Sick sinus syndrome 2
TAAD	Thoracic aortic aneurysm and dissection
TTNtvs	Titin-truncating variants
VUSs	Variants of unknown significance
