# Supplementary material for: The Role of Next-Generation Sequencing in the Management of Asymptomatic, Young Slovenian Athletes for Distinction Between Athlete’s Heart and Cardiomyopathy
Source: Biomedicines. 2026 Jul 2;14(7):1505. doi: 10.3390/biomedicines14071505 (PMC13405593; doi:10.3390/biomedicines14071505)
Supplement: Supplementary file 1 [file biomedicines-14-01505-s001.zip › biomedicines-4369121-supplementary.pdf]

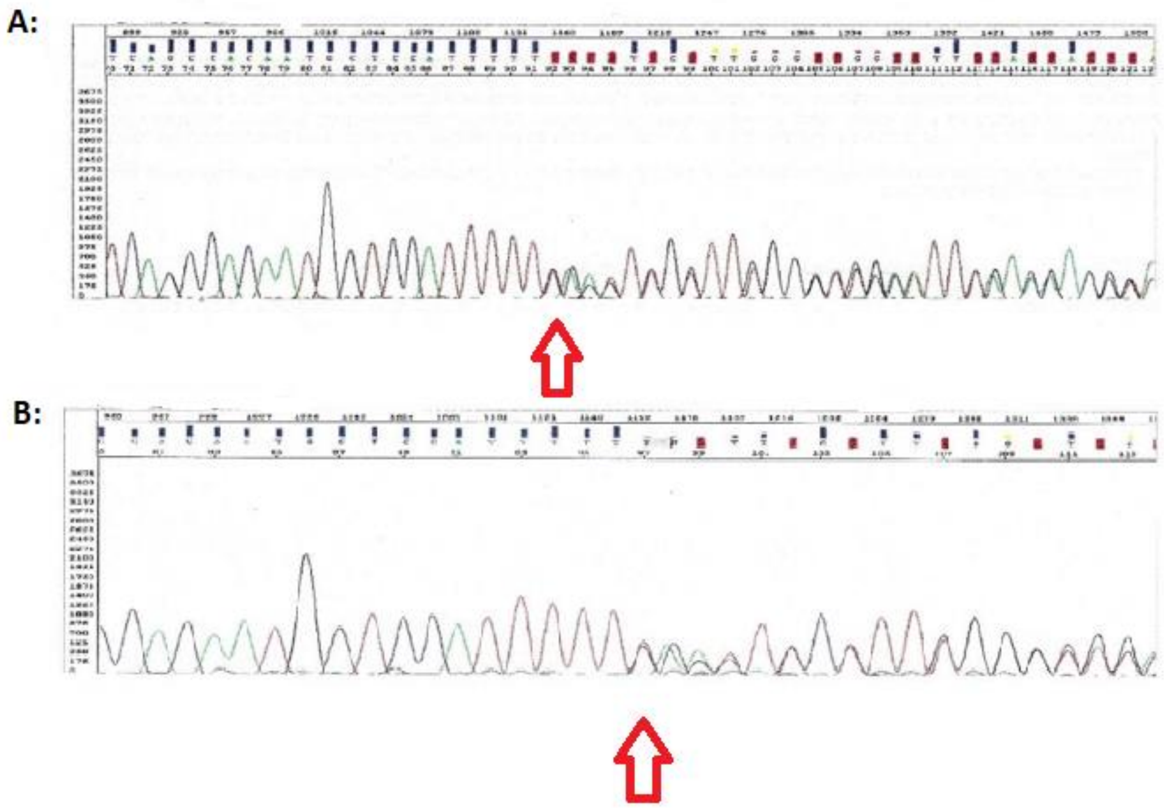

Figure S1. Sanger sequencing - segregation of the variant c.80478dupA in the *TTN* gene (A: athlete 1; B: mother of athlete 1).
